# Fever with lymphadenopathy – Kikuchi Fujimoto disease, a great masquerader: a case report

**DOI:** 10.1186/s13256-017-1521-y

**Published:** 2017-12-16

**Authors:** Chamara Dalugama, Indika Bandara Gawarammana

**Affiliations:** Department of Medicine, University of Peradeniya, Peradeniya, Sri Lanka

**Keywords:** Kikuchi Fujimoto disease, Histiocytic necrotizing lymphadenitis, Fever

## Abstract

**Background:**

Kikuchi Fujimoto disease is an uncommon benign condition of necrotizing histiocytic lymphadenitis commonly seen in East Asian and Japanese populations. It commonly presents with fever, cervical lymphadenopathy, and elevated inflammatory markers. Diagnosis of Kikuchi Fujimoto disease is based on histopathological studies of the involved lymph nodes. The presentation of Kikuchi Fujimoto disease can mimic many sinister conditions including lymphoma. Treatment is mainly supportive provided that accurate diagnosis is made and sinister conditions like lymphoma ruled out.

**Case presentation:**

We report the case of an 18-year-old Sri Lankan Moor woman who presented with fever and cervical lymphadenopathy for 1 month. She had elevated inflammatory markers with high lactate dehydrogenase and ferritin levels. She had an extensive work-up including an excision biopsy of an involved lymph node and bone marrow biopsy. Finally, a diagnosis of Kikuchi Fujimoto disease was based on histopathology of the lymph node and negative bone marrow biopsy.

**Conclusions:**

Although Kikuchi Fujimoto disease is a self-limiting condition, it is a great masquerader which mimics the clinical features of many sinister conditions including tuberculosis, lymphoma, and adult-onset Still’s disease. Early recognition of the disease is of crucial importance in minimizing potentially harmful and unnecessary evaluations and treatments.

## Background

Kikuchi Fujimoto disease (KFD) is an uncommon benign condition of necrotizing histiocytic lymphadenitis commonly seen in East Asian and Japanese populations [1–5]. Although many bacteria, viruses, and autoimmune conditions were attributed to its etiopathogenesis, none of these were consistently associated with the condition [6–14]. KFD commonly presents with fever, cervical lymphadenopathy, and elevated inflammatory markers [15–21]. An elevated lactate dehydrogenase (LDH) level with lymphadenopathy in KFD can lead to diagnostic confusion between KFD and lymphoma for the treating physician [22, 23]. Raised ferritin levels can be associated with KFD. Raised ferritin levels may be due to original disease, co-occurrence of adult-onset Still’s disease (AOSD), or complication of reactive hemophagocytic lymphohistiocytosis (HLH) [24–28]. Diagnosis of KFD is based on histopathological studies of the involved lymph nodes [29, 30]. KFD is a benign self-limiting condition with excellent prognosis. Very few fatalities have been reported. Management is mainly supportive with antipyretics and analgesics. In a few complicated cases, steroids and other immunosuppressive treatment were used successfully [31–37]. It is prudent to follow up patients as a few cases have eventually progressed to systemic lupus erythematosus (SLE) [38].

We present a case of a young 18-year-old woman presenting with fever for 1 month with constitutional symptoms, high inflammatory markers, anemia, and high levels of ferritin. Lymphoma, tuberculosis, or AOSD were considered in the differential diagnosis. However, the histology of the lymph node indicated KFD and she responded well to a short course of steroids. We present this case to emphasize that early recognition of the disease is of crucial importance in minimizing potentially harmful and unnecessary evaluations and treatments and alleviating patients’ agony.

## Case presentation

We report the case of an 18-year-old Sri Lankan Moor woman from the Central Province of Sri Lanka who presented to the Teaching Hospital, Peradeniya with a history of high spiking fevers of 1 month’s duration. She was treated twice by her general practitioner with courses of orally administered co-amoxiclav and azithromycin to which she did not respond. She complained of daily high fevers with drenching sweating. She had severe anorexia and lost 8 kg over the period of 1 month. She had symmetrical inflammatory-type small and large joint arthralgia with morning stiffness for more than 1 hour. She had noticed her neck glands swell during this period. She denied having sore throat, rash, red eyes, or chronic cough. She had no alteration in her bowel habits. On further questioning, she confirmed no prior contact with a known or suspected case of tuberculosis. She was from a middle class Sri Lankan family and a school girl daily travelling from home. She had not had a sexual partner.

On examination she was febrile with a temperature of 37.78 °C (100 °F). She had moderate pallor with no icterus. She had left-side cervical lymph node enlargement with the largest node measuring 2.5 × 1.5 cm in the posterior triangle. Her lymph nodes were discrete, tender, and rubbery in consistency. She had mild ankle edema. Her cardiovascular and respiratory systems were unremarkable. Her abdomen was soft and non-tender with no clinically detectable organomegaly.

Her complete blood count was significant for hemoglobin of 7.4 g/dL with mean corpuscular volume of 74 fL. Her white cell count was 13 × 10^6^/ml with a normal platelet count. A peripheral smear showed normocytic normochromic anemia with marked rouleaux formation. Her erythrocyte sedimentation rate (ESR) was 144 mm in first hour with a C-reactive protein of 60 mg/L. Her renal functions were within range with no protein or cells in urine. Her transaminases were within the normal limits. She had marginally low serum albumin of 35 g/L. A chest radiograph and X-ray of hers hands were unremarkable.

She was empirically started on a broad-spectrum antibiotic after septic screen including urine and blood cultures which were negative subsequently. However, the fact that she had persistent fever spikes warranted further investigations. An ultrasound of her neck confirmed lymphadenopathy and lymph node architecture was preserved. There was no ultrasonic evidence of organomegaly or para-aortic lymphadenopathy. A two-dimensional echocardiogram was normal. Her LDH level was 1254 u/L. Serum ferritin was > 1200 mg/mL. Anti-nuclear factor (ANA) and rheumatoid factor were negative. A peripheral smear for malarial parasite was negative. A Mantoux test was negative.

Two provisional diagnoses were made: considering fever with B symptoms, lymphadenopathy, and high LDH, a provisional diagnosis of lymphoma was made; and considering her fever, elevated blood counts (major criteria), lymphadenopathy (minor criteria) along with an elevated ferritin level, a provisional diagnosis of AOSD was made.

**Fig. 1:**
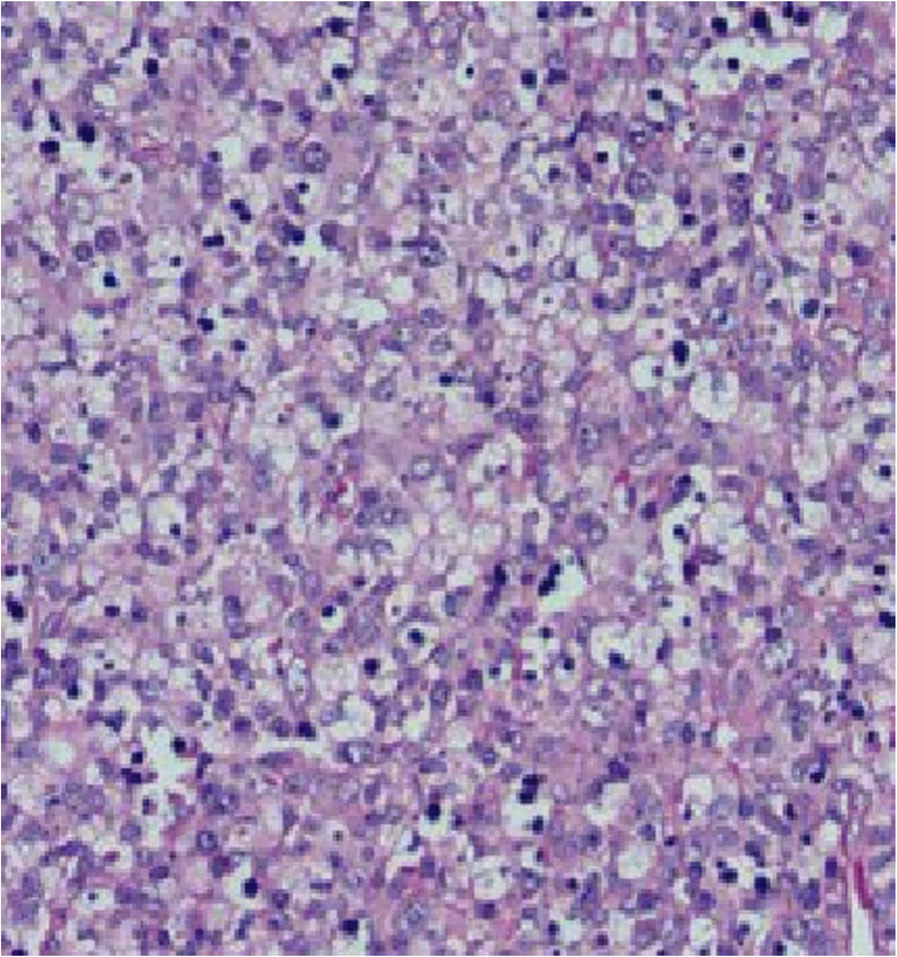
Medium-power view of paracortex showing partially effaced architecture and areas of necrosis, infiltrated with nuclear dust; surrounding tissue shows mononuclear cells with ingested nuclear debris

A cervical lymph node biopsy revealed partially effaced architecture and areas of necrosis, infiltrated with nuclear dust; surrounding tissue shows mononuclear cells (Fig. 1). Histopathological findings were compatible with necrotizing lymphadenitis more in keeping with Kikuchi’s lymphadenitis. Prior to starting steroids she underwent a bone biopsy and subsequently it was found to be a normal marrow.

She was started on orally administered prednisolone 30 mg daily. She was fever free 24 hours after starting steroids and had a marked sense of clinical wellbeing. Currently she is followed up in the medical clinic. At 1 month her ESR had come down to 40 mm in first hour and she was asymptomatic with reduction in the size of cervical lymph nodes. Now she is on tapering off doses of prednisolone.

## Discussion

This case report describes a young woman with pyrexia of unknown origin for 1 month with lymphadenopathy, constitutional symptoms, anemia, and high ferritin level. Many sinister diagnoses including lymphoma, tuberculosis, and AOSD were considered. A diagnosis of KFD was made following a histological examination of lymph node biopsy. She had markedly elevated ferritin levels in the absence of coexisting AOSD or HLH which is a rather uncommon finding compared to typical cases of KFD.

In 1972 Dr Masahiro Kikuchi and Dr Fujimoto presented two cases of lymphadenitis showing focal reticulum cell hyperplasia with nuclear debris and phagocytosis separately [1, 2]. The condition is most frequently found among East Asian and Japanese populations with a slightly female preponderance [3] and it largely affects young adults [4]. Although described internationally, the local disease pattern or incidence had not been well studied. Abeysekara *et al*. described nine cases showing histopathological features of Kikuchi’s disease in Sri Lanka [5]. All patients were female, in the age group of 12 to 30 years, and had fever and lymphadenopathy.

The etiopathogenesis of KFD is not well described in the literature. Although multiple viruses and bacteria are suspected there is no consistent evidence. Bacteria such as *Yersinia enterocolitica* [6], Brucellosis [7], and *Bartonella henselae* [8], and viruses such as Epstein–Barr virus [9], herpes viruses [10], cytomegalovirus [11], parvovirus [12], dengue virus [13], and human immunodeficiency virus [14] were implicated in the etiopathogenesis. None of these were consistently associated with the condition.

The onset of KFD is acute or subacute, evolving during a period of 2 to 3 weeks. The main clinical feature in KFD is unilateral cervical lymphadenopathy [15, 16]. The jugular and posterior cervical group of lymph nodes are more commonly involved [17]. The involvement of other lymph node groups is uncommon. In a retrospective study of 199 patients with KFD by Cheng *et al*., only 2.6% of patients had axillary lymphadenopathy [18]. Generalized lymphadenopathy is far more uncommon. Our patient had unilateral cervical lymphadenopathy with preserved architecture as commonly described.

No diagnostic or specific laboratory testing is available for KFD. Elevated ESR is the most common observation in all the cases reported. Reported hematological findings are leukopenia, neutropenia, lymphocytosis, thrombocytopenia, or anemia [19]. Leukopenia was present in 31.3% of patients in a study of 96 cases by Kwon *et al*. [20]. A few patients had atypical lymphocytes in the peripheral smear [16]. Elevated levels of transaminases are a rare finding [21]. Our patient had normal levels of transaminases and neutrophil leukocytosis. A peripheral smear revealed rouleaux formation, but lymphocytes had normal morphology.

Elevated LDH is a common finding in Kikuchi’s disease and in some it was associated with liver involvement [22, 23]. Our patient had elevated LDH with normal transaminases with fever and B symptoms masquerading as a lymphoma.

Our patient had high serum ferritin of more than 1200 mg/mL. It is rather an uncommon finding in KFD [24]. Considering her fever, elevated blood counts (major criteria), lymphadenopathy (minor criteria) along with an elevated ferritin level, a provisional diagnosis of AOSD was considered in our patient. However, later histopathology of lymph node confirmed the diagnosis of KFD. KFD and AOSD are rare inflammatory conditions with some overlapping features. Toribio *et al*. described a case of rare co-occurrence of KFD and AOSD [25].

KFD is rarely complicated with reactive HLH. A literature review showed that patients with HLH-associated KFD may have higher serum ferritin and LDH levels compared to typical cases of KFD [26–28].

A high ferritin level may indicate the underlying inflammatory condition of KFD, alternative diagnosis of AOSD, rare co-occurrence of KFD with AOSD, or KFD complicated with HLH. In our case, our patient was not very ill and her bone marrow was normal which ruled out the possibility of HLH.

The definitive diagnosis of KFD is made through lymph node excision biopsy and histological examination. Classification of the histopathological changes into three histological types was proposed: proliferative, necrotizing, and xanthomatous types [29]. The absence of granulocytes is also an important feature. Kuo *et al*. suggested that the xanthomatous stage is not the resolving stage of KFD but is a histological variant of KFD [30]. However, it is a challenge for the pathologist to differentiate it from SLE, lymphoma, drug -induced lymphadenopathy, or Kawasaki disease.

KFD is typically a self-limited disease that rarely requires specific treatment and resolves within 1 to 4 months. Cervical lymphadenopathy is benign and self-limiting. Very few cases were reported as fatal, and they were particularly complicated with HLH or neurological manifestations [31]. The treatment is mainly supportive including antipyretics and analgesics, such as paracetamol and nonsteroidal anti-inflammatory drugs (NSAIDS) [32]. Corticosteroids are generally reserved for severe cases, or where supportive measures fail to control symptoms [33]. Other immunosuppressive agents (hydroxychloroquine, cyclosporine, and azathioprine) and immunoglobulin have been used successfully in individual cases [34–37]. The long term follow up and monitoring of patients with KFD for the development of SLE is prudent as there is a slightly increased risk of developing SLE [38].

## Conclusions

Although KFD is a self-limiting condition, it is a great masquerader which mimics the clinical features of many sinister conditions. The clinical features of patients with the final diagnosis of KFD have many provisional diagnoses on presentation. It can be easily mistaken for tuberculosis, lymphoma, AOSD, and so on. Early recognition of the disease is of crucial importance in minimizing potentially harmful and unnecessary evaluations and treatments. Treatment of KFD is mainly supportive. It is prudent to follow up patients as a few cases have eventually progressed to SLE.
